# Driver Drowsiness Detection Based on Steering Wheel Data Applying Adaptive Neuro-Fuzzy Feature Selection

**DOI:** 10.3390/s19040943

**Published:** 2019-02-22

**Authors:** Sadegh Arefnezhad, Sajjad Samiee, Arno Eichberger, Ali Nahvi

**Affiliations:** 1 Institute of Automotive Engineering, Mechanical Engineering Department, Graz University of Technology, Graz 8010, Austria; s.arefnezhad@tugraz.at (S.A.); arno.eichberger@tugraz.at (A.E.); 2 Mechanical Engineering Department, K.N. Toosi University of Technology, Tehran 19991-43344, Iran; nahvi@kntu.ac.ir

**Keywords:** adaptive neuro-fuzzy inference system (ANFIS), driver drowsiness detection, feature selection, particle swarm optimization (PSO)

## Abstract

This paper presents a novel feature selection method to design a non-invasive driver drowsiness detection system based on steering wheel data. The proposed feature selector can select the most related features to the drowsiness level to improve the classification accuracy. This method is based on the combination of the filter and wrapper feature selection algorithms using adaptive neuro-fuzzy inference system (ANFIS). In this method firstly, four different filter indexes are applied on extracted features from steering wheel data. After that, output values of each filter index are imported as inputs to a fuzzy inference system to determine the importance degree of each feature and select the most important features. Then, the selected features are imported to a support vector machine (SVM) for binary classification to classify the driving conditions in two classes of drowsy and awake. Finally, the classifier accuracy is exploited to adjust parameters of an adaptive fuzzy system using a particle swarm optimization (PSO) algorithm. The experimental data were collected from about 20.5 h of driving in the simulator. The results show that the drowsiness detection system is working with a high accuracy and also confirm that this method is more accurate than the recent available algorithms.

## 1. Introduction

### 1.1. Driver Drowsiness Detection

Drowsiness detection is an important factor for road safety, in manual driving as well as in future semi-automated driving. In manual driving, about 20 to 30% of fatal road accidents are reported to be attributable to driver drowsiness [1,2]. In SAE level 3 automated driving, drivers will be allowed to do a secondary task, but the automation system has to hand-over vehicle guidance whenever it cannot manage the situation. A hand-over to a drowsy driver will eventually not be manageable, so a restriction of vehicle automation to alert drivers detected by a reliable drowsiness detection system could be an option for oncoming SAE level 3 systems.

Two general strategies could be considered to detect driver drowsiness: intrusive and non-intrusive. In intrusive methods, the drowsiness state is analyzed using processing of physiological outputs such as electroencephalographic (EEG) and electrooculographic (EOG) information [3]. High performance for drowsiness detection could be obtained using intrusive methods but driver movements can negatively affect the reliability of the designed system. Non-intrusive methods provide an estimation for drowsiness using facial features of drivers [4,5] or vehicle-based measures [6]. On one hand, these methods have some shortcomings. As an example, different lighting conditions can have disrupting effects on the detection performance. On the other hand, vehicle-based measures reflect the driving behavior, and therefore, can provide an acceptable estimation for drowsiness detection in an unobtrusive way [7]. Gas and brake pedal inputs, changes in vehicle speed, steering wheel angle and velocity signals and lateral distance of the vehicle from center of the line are typically used as vehicle-based measurement to detect the drowsiness [8].

Steering wheel data have been employed in several previous studies in this application. For example, Li et al. in [9] presented a method for online drowsiness detection in drivers based on extracted approximate entropy from Steering Wheel Angle (SWA) signals. Extracted approximate entropy feature was linearized using adaptive piecewise linear fitting and the wrapping distance between linear feature series was used in binary classifier to determine the drowsiness state. Frequency and time-frequency features of SWA were extracted by applying Short Time Fourier Transform (STFT), and Wavelet Transform (WT) in [10] for drowsiness detection. Meng et al. in [8] used a driving simulator to collect eleven features related to steering wheel and applied multivariate analysis of variance to find parameters that had significant correlation with the level of drowsiness. Using this method, the numbers of parameters were reduced to five features. This paper employs steering angle and velocity data for driver drowsiness detection as an application in order to testify the performance of the proposed feature selection method. 

### 1.2. Feature Selection

Feature selection is a method to select a subset of features from the multidimensional data space in order to improve the accuracy of classifiers, reduce the computational burden in classification process and better understanding of the data in machine learning applications [11]. Real-world data set mostly contains redundant and dependent variables that cannot provide extra information about the class labels. Moreover, some of the features usually do not have correlation to the class labels and can produce bias noise for the classifiers and reduce its classification performance [12].

Feature selection is an active research field and has been used in different applications such as bioinformatics data mining [13], image processing [14], text classification [15] and fault diagnosis in mechanical systems [16,17]. Different feature selection techniques have been proposed by researchers that can be generally classified in three categories: the filter approach, the wrapper approach and the embedded approach [18]. The filter method is based on association between the feature and the class label and it is independent of learning models [19]. In contrast, in the structure of wrapper approach, a learning model is employed and features are selected with the aim of performance improving of the learning model [11]. Embedded methods have been proposed to reduce the computation time in wrapper methods and feature selection is a part of training process in these methods. In other words, embedded methods specify which features provide the best accuracy while the model is being created [20].

Fuzzy inference systems have been used in the structure of feature selection methods [21]. Fuzzy logic can be useful for solving the multi-objective optimization problem of feature selection process to maximize the model accuracy and minimize the number of used features and avoid weighting of different goals in this kind of optimization problems [22]. Cateni et al. in [23] designed a method for feature selection by combining four different filter methods using fuzzy logic to specify the importance degree of each feature. Features that have an importance degree more than a predefined threshold value could be exploited as inputs to classifiers. In order to adjust the parameters of fuzzy system, Particle Swarm Optimization (PSO) has been wieldy used by researchers for example in applications of freight volume forecasting [24] and heating system planning problem [25].

In the previous fuzzy feature selection methods, predefined parameters of fuzzy inference systems are constant and independent to the dynamical behaviors of data. In this paper a new method based on neuro-fuzzy logic has been proposed which is a combination of filter methods and neuro-fuzzy-wrapper method. Adaptive training of the parameters in the fuzzy rule-base considers different dynamical changes in the dataset. In the first step, four different filter methods (Fisher, T-test, Correlation and Mutual information) have been exploited used to determine the importance index of each feature, then a fuzzy inference system has been designed and four filter indexes have been used as inputs of the fuzzy system to determine the final importance degree of each feature. Features which have importance degrees more than a predefined threshold value have been employed in the Support Vector Machine (SVM) binary classifier. Classifier accuracy has been used as a feedback for the adjustment of parameters in the fuzzy membership functions. PSO as an evolutionary optimization algorithm, has been exploited to train Adaptive Neuro-Fuzzy Inference System (ANFIS). In order to evaluate the proposed method, a real-world data set related to drowsiness detection in drivers has been used. Experimental results show that proposed method has a better accuracy than non-adaptive fuzzy feature selection methods. 

This paper is organized as follows: Section 2 describes the proposed method for feature selection. Section 3 presents used data set and our driving simulator for data acquisition. Results have been presented in Section 4 and finally, this paper has been concluded in Section 5.

## 2. Materials and Methods

**Figure 1:**
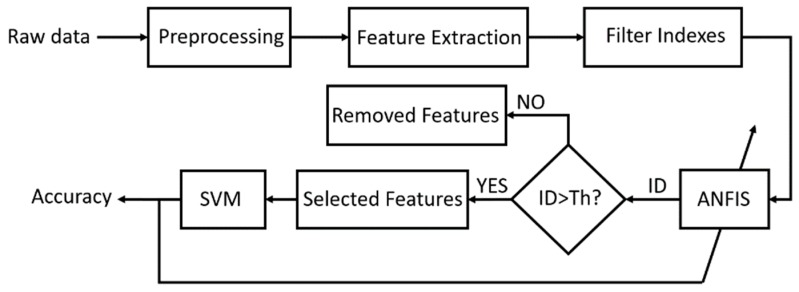
Scheme of the proposed approach procedure; ID and Th mean Importance Degree and its Threshold, respectively.

The proposed approach for feature selection is based on combination of filter and wrapper methods using ANFIS. Using parameter adjustment algorithms, ANFIS can help to select the best features based on dynamical behaviors of raw data. Figure 1 shows the structure of the proposed method. In the following sections, different parts of the proposed method have been explained.

### 2.1. Preprocessing

Steering wheel angle signal is dependent to the road geometry and curvature. In the straight sections of the road, steering wheel signals only consist of lane-keeping adjustments of steering by drivers. In contrast, steering signals in curved sections consist of road geometry effects. As the test scenario includes both straight and curved sections, the effect of road curvature on steering signal should be removed in sliding windows to detect the drowsy states in drivers [26]. This was performed by applying the following equations:

(1) $$M_{\theta }=\frac{1}{W}\overset{n_{l}+w-1}{\underset{k=n_{l}}{\sum }}\theta \left(k\right)$$

(2) $$\theta *\left(k\right)=\theta \left(k\right)-M_{\theta }$$

where, W is the length of sliding window, $n_{l}$ is the first point (left side) of the window and $M_{\theta }$ is the average of steering angle, θ, in the sliding window which has been subtracted from raw signal to obtain the θ*(k) which is the preprocessed signal.

**Figure 2:**
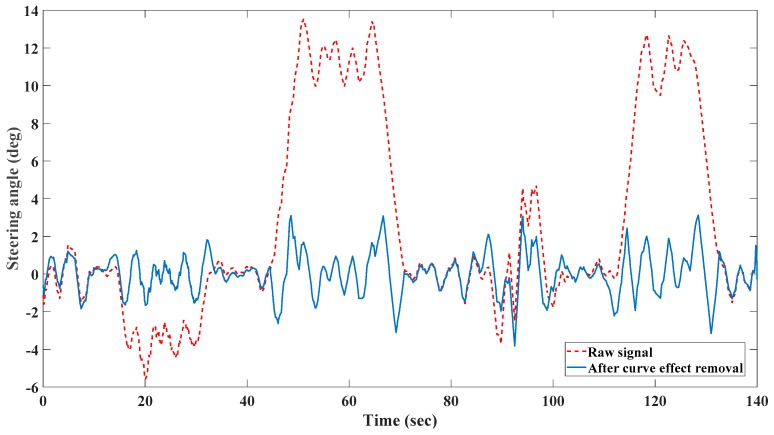
Road curve effect removal.

Since there is no specific window size mentioned in literatures and in order to select the optimal length of the window for our data, different window lengths have been tested and the best performance of the system has been obtained by three-second window. We also considered 50 percent of overlap (1.5 s) for each two successive windows to retain the dynamics behavior of input data. The same window length and overlap percentage have been also used to extract the features from signals in Section 2.2. Figure 2 shows the result of road curve effect removal on steering angle data. According to this figure, the driver is trying to follow the two left turns (the steering angles are positive) based on the raw signal (dashed line) and the effect of the curvy part of the road has been cancelled in the preprocessed signal (solid line).

### 2.2. Feature Extraction

Steering signal contains different information of driving behavior in time and frequency domains. Before feature selection, a sliding window with the length of three seconds (180 samples) and 1.5 s (90 samples) of overlap has been applied on the signal. Thirty-six features have been extracted form steering wheel angle and velocity signals in time and frequency domains. These features have been selected based on some previous works on drowsiness detection of drivers using steering wheel signals [10,27,28]. 

Time domain features used in the model were range, standard deviation, energy, Zero Crossing Rate (ZCR), first to third quartiles, Katz Fractal Dimension (KFD), skewness, kurtosis, Sample Entropy (SamEn), and Shannon Entropy (ShEn). Frequency domain features were frequency variability, Spectral Entropy (SpEn), spectral flux, Center of Gravity of Frequency (CGF), dominant frequency and average value of power spectral density. Extracted features have been presented in Table 1 and Table 2, where superscript a refers to features from steering angle signal and superscript v demonstrates features extracted from steering velocity signal. Extracted features have been normalized between 0 and 1 using their minimum and maximum values.

### 2.3. Filter Method Indexes

Consider the feature matrix and classes output as given in Equations (3) and (4), respectively:

(3) $$X_{M\times N}={\left[\begin{array}{ccc} x_{1} & x_{2} & \begin{array}{cc} \ldots & x_{M} \end{array} \end{array}\right]}^{T}$$

(4) $$Y_{N\times 1}={\left[\begin{array}{ccc} y_{1} & y_{2} & \begin{array}{cc} \ldots & y_{N} \end{array} \end{array}\right]}^{T}$$

where, $x_{i}\;(i=1,2,\ldots ,M)$ is a N×1 feature vector, $y_{j}\;(j=1,2,\ldots ,N)$ is a binary class label, $y_{j}\in \left\{0,1\right\}$, M is the number of extracted features from input signals and N is the number of samples for each feature. The aim of the proposed method is selecting the best features to have the highest classifier accuracy for predicting the class label. In the proposed method, four different filter indexes have been derived for each feature. These indexes include Fisher, Correlation, T-test and Mutual Information and have been briefly explained as follows:

#### 2.3.1. Fisher Index

Fisher index has been widely used by researchers for feature selection and it could be calculated for each feature independently of other features [29]. This index could be calculated by Equation (5):

(5) $$F\left(i\right)=\left|\frac{\mu_{1}\left(x_{i}\right)-\mu_{0}\left(x_{i}\right)}{\sigma_{1}^{2}\left(x_{i}\right)-\sigma_{0}^{2}\left(x_{i}\right)}\right|$$

where $\mu_{j}\left(x_{i}\right)$ and $\sigma_{j}^{2}\left(x_{i}\right)$ are the mean value and standard deviation in the samples belonging to the two classes, respectively.

#### 2.3.2. Correlation Index

Pearson correlation coefficient is a simple filter index for feature selection [30]. It is calculated by (6):

(6) $$R\left(i\right)=\frac{cov\left(x_{i},Y\right)}{\sigma \left(x_{i}\right)\times \sigma \left(Y\right)}$$

where, $cov\left(x_{i},y\right)$ is the covariance between the *i*-th feature $x_{i}$ and class label Y, $\sigma \left(x_{i}\right)$ is standard deviation of the *i*-th feature and σ(Y) is standard deviation of the class label.

#### 2.3.3. T-test Index

T-test index is used to determine specific statistical difference between two variables [31]. This index is calculated using Equation (7):

(7) $$T\left(i\right)=\frac{\left|\mu_{1}\left(x_{i}\right)-\mu_{0}\left(x_{i}\right)\right|}{\sqrt{\frac{\sigma_{1}^{2}\left(x_{i}\right)}{n_{1}}+\frac{\sigma_{0}^{2}\left(x_{i}\right)}{n_{0}}}}$$

where $n_{0}$ and $n_{1}$ are the number of observations belonged to two different classes.

#### 2.3.4. Mutual Information Index

Mutual information is a method of measuring the amount of information that could be obtained for one random variable through the other random variable [32]. This index intends to calculate the relevancy between each feature and class label using mutual information and is calculated using Equation (8):

(8) $$I\left(x_{i}\right)=\underset{x_{i}}{\sum }\underset{Y}{\sum }p\left(x_{i}.Y\right)\times \log\left(\frac{p\left(x_{i},Y\right)}{p\left(x_{i}\right)\times p\left(Y\right)}\right)$$

where $p\left(x_{i}\right)$, p(Y) and $p\left(x_{i},Y\right)$ are Probability Density Function (PDF) for the *i*-th feature, PDF for class label outputs and joint PDF between $x_{i}$ and Y, respectively.

### 2.4. Fuzzy Inference System

**Figure 3:**
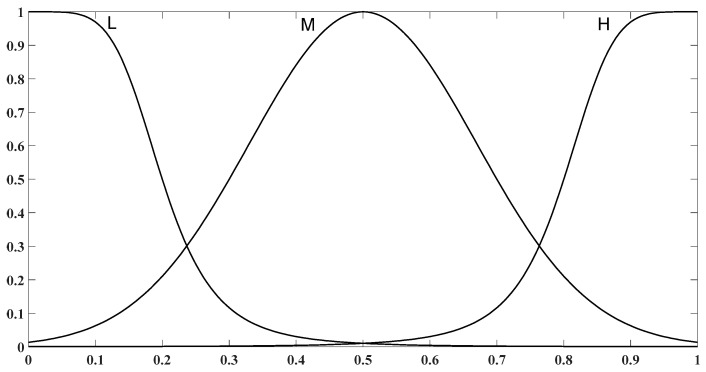
Initial membership functions for all of fuzzy system inputs.

In order to combine advantages of four used filter feature selection methods, fuzzy logic and adaptive adjustment of its parameters have been exploited. Every fuzzy system consists of four main parts: fuzzifier, fuzzy rule-base, inference engine and defuzzifier. Gaussian membership functions as given in (9) have been selected for fuzzification process:

(9) $$f_{i}^{j}\left(h_{j}\right)=\exp\left(-\frac{1}{2}{\left(\frac{h_{j}-c_{i}^{j}}{s_{i}^{j}}\right)}^{2}\right)$$

where $h_{j}$ is the input to the fuzzy system. In this type of membership functions, mean, c and standard deviation, s are nonlinear parameters that should be adjusted by optimization algorithms. Three Membership Functions (MFs), Low (L), Medium (M) and High (H), have been considered for each input. These membership functions have been shown in Figure 3. Takagi-Sugeno inference type by singleton consequences in fuzzy rules has been used in the fuzzy rule-base part. By using this inference type, deffuzification process has been avoided [33]. This fuzzy rule-base has 81 rules and three singleton consequences have been considered for Importance Degree (ID) in each fuzzy rule. The l-th rule of the rule base has been described in Equation (10):

(10) $$l:If\;F\;is\;A_{1}^{l}\;and\;R\;is\;A_{2}^{l}\;and\;T\;is\;A_{3}^{l}\;and\;I\;is\;A_{4}^{l}\;then\;ID=\alpha^{l}$$

where, F, R, T and I are Fisher, correlation, T-test and mutual information indexes, respectively, and $A_{1}^{l}$ to $A_{4}^{l}$ are MFs for inputs and could gain each of three different states of L, M and H. ID is the determined importance degree obtained from the fuzzy rule, and $\alpha^{l}$ which has three different possible values of 0, 0.5 and 1, is fuzzy singleton consequence. Threshold of 0.5 for ID has been used to select the final features to be inputted to the SVM classifier.

### 2.5. ANFIS Training by PSO Algorithm

PSO is an evolutionary optimization method that solve the optimization problems by improving the candidate solutions. In this optimization method, candidate solutions may fly in parameter search space to find the best solutions using best performances of their neighbor particles [24,34]. In other words, each individual particle flies in the search space with a dynamically adjusted velocity. This adjustment is based on flying experience of each particle and its neighbors.

PSO has three different main parts. The first part is the momentum for each solution to continue its current direction in the search space. The second part is the personal best for each particle in its memory. Finally, the third part is the global best for the whole of population. In this part, the position of the neighbor particles has been considered to obtain the global best solution for the optimization problem. In this paper, the half of square error, as given in Equation (11), is considered as objective function for optimization problem and adjusting the parameters of ANFIS:

(11) $$J\left(i\right)=\frac{1}{2}{\left[\hat{y}\left(i\right)-y\left(i\right)\right]}^{2}$$

where, $\hat{y}\left(i\right)$ is the estimated binary value for KSS. Details of PSO algorithm have been explained in the Appendix A and the parameters of PSO are listed in Table 3.

### 2.6. Support Vector Machine Classifier

In order to classify the driver states in two classes of drowsy and alert, binary SVM classifier has been applied. Using this classifier, the performance of proposed method for real world data set can be evaluated and compared with other feature selection methods. SVM is a training algorithm for leaning classification and regression rules from data set.

This method is based on obtaining optimal boundary of two sets for classifying the data set. This classification method is a robust algorithm; therefore, it is suitable for noisy and real world data set. This classification method can use different kernel functions to be applicable in nonlinear data space without explicitly requiring a nonlinear algorithm. The details and formulation of the SVM have been described in [35].

## 3. Dataset and Experimental Setup

**Figure 4:**
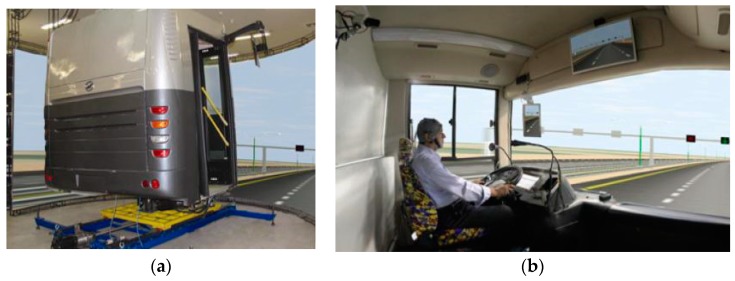
Driving simulator: (**a**) External view; (**b**) Internal view.

Experiments were performed using a bus driving simulator (BI301Semi) at the K. N. Toosi University of Technology. In this simulator, three high-resolution 3D video projectors have been employed to produce a field of view of 180°. In order to simulate road conditions, several pneumatic actuators have been exploited and an electric force feedback has also been used to provide steering wheel torque [36]. Figure 4a,b show the external and internal views of the used driving simulator, respectively.

**Figure 5:**
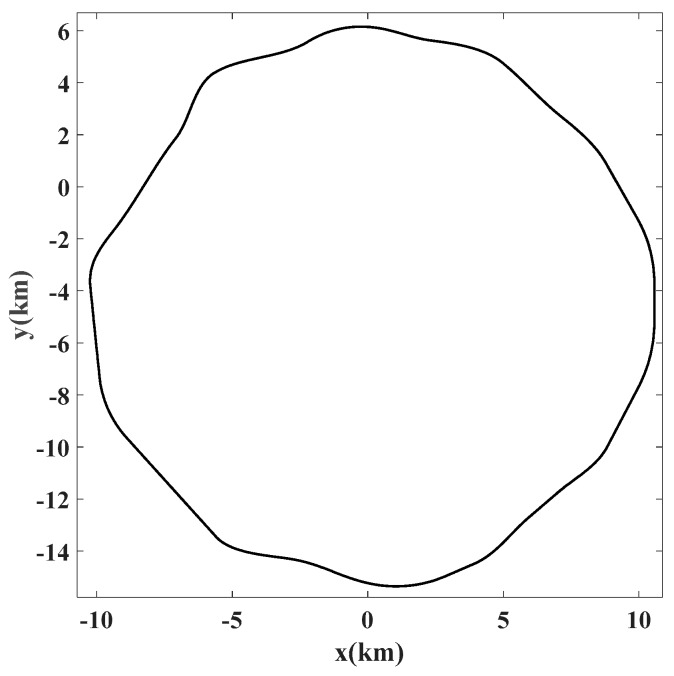
Test track for driving tests.

A total of 39 bus drivers from a variety of driving professions completed the study. The experimental tests were conducted around 2:00 PM until the driver was too drowsy to continue and the overall time of driving tests was about 53 h. After checking the collected data, only driving test that had the whole KSS range from 1 to 9 have been considered to assess the drowsiness level. Selected data set has about 20 h and 36 min of collected variables. Steering wheel angle signal with the sampling rate set to 60 Hz has been gathered to assess the drowsiness. The test track was a closed circular with several sinusoidal smooth curves all through the path. The test track has been presented in the Figure 5. In order to assess the drowsiness level of drivers, binary version of Karolinska Sleepiness Scale (KSS) [7,37] has been employed. Driving periods that were corresponded to KSS values from one to six have been used to define the alert class and KSS values of eight and nine defined the drowsy class. In order to obtain a better separation between two binary classes, samples with KSS value of seven have been ignored in the proposed method.

## 4. Results and Discussion

This section presents the experimental results obtained from applying proposed method on driving tests. Proposed neuro-fuzzy feature selection method as well as each single filter methods that have been explained in pervious sections have been applied on driving simulator dataset to detect the drowsiness in bus drivers. Performance of the proposed method has been evaluated using following indexes:

(1) True Positive (TP): number of drowsy states that correctly classified as drowsy;
(2) True Negative (TN): number of awake states that correctly identified as awake;
(3) False Negative (FN): number of drowsy states that incorrectly identified as awake;
(4) False Positive (FP): number of awake states that incorrectly identified as drowsy;

Using the above indexes, accuracy percentage of each method is calculated with Equation (12):

(12) $$Accuracy=\frac{TP+TN}{TP+FP+TN+FN}\times 100$$

The mentioned indexes can be used in a confusion matrix as shown in Table 4 to evaluate the performance of the proposed method. 

The FP is defined as the number of awake states that are incorrectly identified as drowsy, hence it only describes the situation of false alarming and does not put the driver in dangerous conditions, while FN is the number of drowsy states that are incorrectly identified as awake and can cause risky situations. As in can be seen, the probability percentage of these situations is less than 1.5%, which is acceptable. 

**Figure 6:**
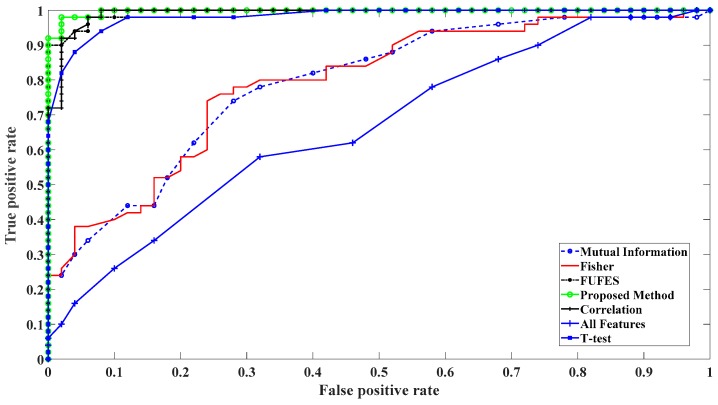
Receiver Operating Characteristic (ROC) curve for evaluation of classifier performance with different feature selection methods.

In order to evaluate the proposed method, its accuracy has been compared with other features selection methods in Table 5. Our dataset has been used to implement on proposed method in [23], where the parameters of fuzzy membership functions were constant. Table 5 demonstrates that our method outperforms the suggested method in [23]. Receiver Operating Characteristic (ROC) has been also applied to compare the results of the proposed method with other feature selection algorithms (Figure 6). The Area Under this Curve (AUC) is between 0 and 1 and AUC with the maximum value corresponds to a perfect classifier. Figure 6 shows that the proposed feature selection algorithm can improve the classifier performance. When all of the features have been used as classifier inputs, redundancy between extracted features can increase the false positive rate and AUC is the lowest value in comparison with other methods. Accuracy and AUC of the different methods have been compared in Table 5. 

Results show that adjustment of the parameters of fuzzy system can outperform FUzzy FEature Selection (FUFES) method when lower number of features have been applied as classifier inputs. Moreover, our method needs lower number of selected features to have a better accuracy for SVM classifier. According to Table 5, T-test index and the proposed method selected the same number of features and three of them are similar but other two different features make about 8 percent of accuracy difference. Although Correlation index provides an acceptable accuracy for classification, it needs 25 features which increases the computation time.

Performance of the proposed system has been compared with previously reported non-invasive drowsiness detection systems in Table 6. This comparison shows that adaptive selection of input features outperforms the classification accuracy. According to the Table 6, it is might be more significant to use nonredundant features for classification than employing different vehicle variables for drowsiness detection in drivers. For example, in [38] Steering wheel angle, lateral displacement, speed, eye blinking and pupil diameter have been exploited in Multilevel Ordered Logit (MOL) to detect the drowsiness, while our method only used steering angle and steering velocity and outperforms the results of [38] about 29%.

**Figure 7:**
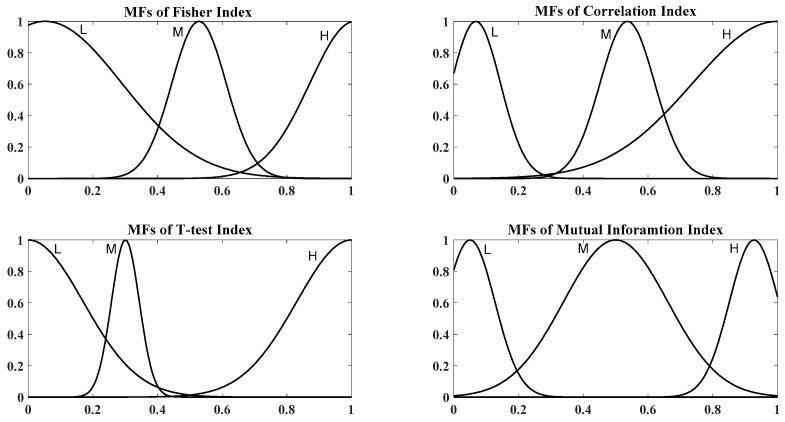
Final membership functions; upper left: Fisher index, upper right: Correlation index, lower left: T-test index, and lower right: Mutual information index.

PSO training of ANFIS made parameter adjustment of fuzzy membership function and their final shapes to be different from their initial. Figure 7 demonstrates the final obtained membership functions for each filter index. Comparison between final and initial membership functions shows that PSO changes both of the parameters of average and standard deviation to decrease the cost function value.

## 5. Conclusions

This study presented a novel method for feature selection using neuro-fuzzy systems and its application in driver drowsiness detection. This method was based on the idea of combining filter and wrapper feature selection methods to improve the performance in comparison with the results when each of the filter and wrapper methods has been employed individually. This combination was performed in the structure of the designed ANFIS. Four different filter indexes were calculated for each feature and used as inputs to ANFIS to produce importance degree of each feature. In order to train ANFIS parameters, PSO as an evolutionary optimization method was exploited. Finally, most important features based on ANFIS output were selected to be inputted to the binary SVM classifier in order to classify the driving state into two classes of alert and drowsy. Steering wheel angle and velocity signals of performed experimental tests in driving simulator were used as vehicle-based measurements to detect the drowsiness and 36 features in time and frequency domains were extracted. Final results showed that the proposed method can produce higher accuracy by using less features than other feature selection methods. 

The advantages and innovations of the proposed drowsiness detection system include (1) adaptive selection drowsiness-related features based on the dynamic behavior of steering wheel, (2) applying ANFIS to improve fuzzy feature selection by fuzzy parameter adjustment and (3) exploiting the advantages of four different filter feature selection methods to improve the reliability of the classification results. In order to improve the suggested method following tasks are proposed which are the goals of the future studies: (1) using of more vehicle-based data such as lateral velocity and lane deviation from the center line, (2) exploiting of electroencephalogram (EEG) signals to extract a more reliable ground truth for drowsiness levels, (3) applying new machine learning algorithms such as deep neural networks to personalize the designed system based on individual driving behavior of each driver, and (4) prediction of the critical level of drowsiness to aware the driver before driving malfunctions. 

## Figures and Tables

**Table 1:** Extracted time domain features from steering angle and velocity signals.

Index	Time Domain Features	Descriptions
$I_{1}^{a},I_{1}^{v}$	Range	Difference between minimum and maximum of signal
$I_{2}^{a},I_{2}^{v}$	Standard Deviation	Dispersion of the data around mean value
$I_{3}^{a},I_{3}^{v}$	Energy	Sum of the square of signal magnitude
$I_{4}^{a},I_{4}^{v}$	Zero Crossing Rate (ZCR)	Number of steering or steering velocity direction changes per second
$I_{5}^{a},I_{5}^{v}$	First Quartile	Middle number between the smallest number and the median of the signal in sliding window
$I_{6}^{a},I_{6}^{v}$	Second Quartile	Median of the signal in the sliding window
$I_{7}^{a},I_{7}^{v}$	Third Quartile	Middle value between the median and the highest value of the signal in sliding window
$I_{8}^{a},I_{8}^{v}$	Katz Fractal Dimension (KFD)	An index for characterizing fractal patterns or sets by quantifying their complexity as a ratio of the change in detail to the change in scale.
$I_{9}^{a},I_{9}^{v}$	Skewness	A measure for signal similarity
$I_{10}^{a},I_{10}^{v}$	Kurtosis	Measure of tailedness of the probability distribution of a random variable
$I_{11}^{a},I_{11}^{v}$	Sample Entropy (SamEn)	Complexity of signal in time domain based on distance in embedding dimension
$I_{12}^{a},I_{12}^{v}$	Shannon Entropy (ShEn)	Complexity of signal in time domain based on probability function

**Table 2:** Extracted frequency domain features from steering angle and velocity signals.

Index	Frequency Domain Features	Descriptions
$I_{13}^{a},I_{13}^{v}$	Frequency Variability	Variance of the frequency in the defined frequency band
$I_{14}^{a},I_{14}^{v}$	Spectral Entropy (SpEn)	Complexity of signal in frequency domain
$I_{15}^{a},I_{15}^{v}$	Spectral Flux	Difference in the spectrum between two adjacent frames
$I_{16}^{a},I_{16}^{v}$	Center of Gravity of Frequency (CGF)	Spectral centroid of the signal
$I_{17}^{a},I_{17}^{v}$	Dominant Frequency	The frequency that has maximum value of the Power Spectral Density (PSD)
$I_{18}^{a},I_{18}^{v}$	Average Value of PSD	Mean value of PSD of a sliding window in frequency domain

**Table 3:** Parameters of PSO.

Parameter	Notation	Value
Cognitive coefficient	$C_{1}$	2
Social coefficient	$C_{2}$	2
Number of population	$N_{P}$	50
Inertia weight	W	0.95
Random matrices ^1^	$R_{1}$, $R_{2}$	Not constant

^1^ Random matrices in PSO are diagonal matrices that nonzero elements are uniformly distributed in the unit interval.

**Table 4:** Confusion matrix for proposed method.

		True classes	
		Awake	Drowsy
**Estimated classes**	Awake	TN = 24212	FN = 538
	Drowsy	FP = 814	TP = 9515
	Samples	25026	10053

**Table 5:** Obtained classification accuracy and selected features for each feature selection method.

Method	AUC	Accuracy	No. Selected Features	Selected Features
All features	0.71	88.39	36	All
Fisher	0.79	89.73	6	$I_{3}^{a}$, $I_{11}^{a}$, $I_{15}^{a}$, $I_{10}^{v}$, $I_{11}^{v}$, $I_{17}^{v}$
T-test	0.85	90.21	5	$I_{4}^{a}$, $I_{11}^{a}$, $I_{10}^{v}$, $I_{11}^{v}$, $I_{17}^{v}$
Correlation	0.95	96.47	25	$I_{1}^{a}$, $I_{2}^{a}$, $I_{5}^{a}$, $I_{6}^{a}$, $I_{7}^{a}$, $I_{9}^{a}$, $I_{10}^{a}$, $I_{11}^{a}$, $I_{12}^{a}$, $I_{13}^{a}$, $I_{14}^{a}$, $I_{16}^{a}$, $I_{18}^{a}$, $I_{1}^{v}$, $I_{2}^{v}$, $I_{3}^{v}$, $I_{5}^{v}$, $I_{6}^{v}$, $I_{8}^{v}$, $I_{9}^{v}$, $I_{10}^{v}$, $I_{11}^{v}$, $I_{13}^{v}$, $I_{16}^{v}$, $I_{18}^{v}$
Mutual information	0.78	88.12	6	$I_{4}^{a}$, $I_{6}^{a}$, $I_{12}^{a}$, $I_{11}^{v}$, $I_{15}^{v}$, $I_{17}^{v}$
FUzzy FEature Selection (FUFES)	0.95	96.41	10	$I_{1}^{a}$, $I_{2}^{a}$, $I_{4}^{a}$, $I_{9}^{a}$, $I_{10}^{a}$, $I_{11}^{a}$, $I_{12}^{a}$, $I_{17}^{a}$, $I_{14}^{v}$, $I_{16}^{v}$
Adaptive neuro-fuzzy feature selection	0.97	98.12	5	$I_{3}^{a}$, $I_{11}^{a}$, $I_{4}^{v}$, $I_{10}^{v}$, $I_{11}^{v}$

**Table 6:** Comparison the accuracy of the proposed method with the reported results of non-invasive drowsiness detection system in previous studies.

Study	Method	Used variables	Accuracy
Krajewski et al., 2009 [39]	Ensemble classification using time domain, frequency domain and state space features	Steering wheel angle, lane deviation and pedal movement	86.1
McDonald et al., 2012 [40]	Random forest algorithm	Steering wheel angle	79
Samiee et al., 2014 [27]	Weighted output of three trained neural networks by used variables	Steering wheel angle, lateral displacement and eye blinking	94.63
Wang and Xu, 2016 [38]	Multilevel ordered logit (MOL) modeling using driver behavior and eye features metrics	Steering wheel angle, lateral displacement, speed, eye blinking and pupil diameter	68.40
Li et al., 2017 [9]	Warping distance between linearized approximate entropy in sliding windows	Steering wheel angle	78.01
Proposed study	Adaptive neuro-fuzzy feature selection with SVM classifier	Steering wheel angle and steering wheel velocity	98.12

## Appendix A

The steps of PSO algorithm are the following:

1. Initialization. For each of the N particles:
  (a) Initialize the position, $x_{j}\left(0\right)\;\forall i\in 1:N$
  (b) Initialize the particles best position to its initial position, $P_{i}\left(0\right)=x_{i}\left(0\right)$
  (c) Calculate the performance of each particle and if $f\left(x_{j}\left(0\right)\right)\geq f\left(x_{i}\left(0\right)\right)\;\forall i\neq j$ initialize the global best as *g* = *x_j_*(0)
2. Optimization loop:
  (a) Update the particle velocity according to (A1)
    (A1) $$v_{i}\left(k+1\right)=wv_{i}\left(k\right)+c_{1}\left(P_{i}-x_{i}\left(t\right)\right)R_{1}+c_{2}\left(g-x_{i}\left(t\right)\right)R_{2}$$
  (b) Update the particle position according to (A2)
    (A2) $$x_{i}\left(k+1\right)=x_{i}\left(k\right)+v_{i}\left(k+1\right)$$
  (c) Evaluate the performance of the particle: $f\left(x_{i}\left(k+1\right)\right)$
  (d) If $f\left(x_{i}\left(k+1\right)\right)\geq f\left(P_{i}\right)$, update personal best: $P_{i}=x_{i}\left(k+1\right)$
  (e) If $f\left(x_{i}\left(k+1\right)\right)\geq f\left(g\right)$, update global best: $g=x_{i}\left(k+1\right)$
3. At the end of the iterative process, the best solution is the final g.
